# IFN-γ mediates graft-versus-breast cancer effects via enhancing cytotoxic T lymphocyte activity

**DOI:** 10.3892/etm.2014.1760

**Published:** 2014-06-05

**Authors:** QIANJIE ZHAO, LINGLING TONG, NINGNING HE, GUOWEI FENG, LIANG LENG, WEIJUN SUN, YANG XU, YUEBING WANG, RONG XIANG, ZONGJIN LI

**Affiliations:** 1 Department of Pathophysiology, School of Medicine, Nankai University, Ministry of Education, Tianjin 300071, P.R. China; 2 Key Laboratory of Bioactive Materials, College of Life Science, Nankai University, Ministry of Education, Tianjin 300071, P.R. China

**Keywords:** graft-versus-tumor, breast cancer, cytotoxic T lymphocytes, interferon-γ, granzyme B

## Abstract

Previous studies have demonstrated the beneficial effect of graft-versus-tumor (GVT) following hematopoietic stem cell transplantation (HSCT) on the incidence of leukemia relapse and the overall survival rate of patients with leukemia; however, detailed mechanisms underlying the effects GVT exhibits on solid tumors following allogeneic HSCT are yet to be elucidated. The aim of the present study was to investigate the immune mechanism underlying the effect of interferon (IFN)-γ on GVT following allogeneic HSCT in breast cancer therapy. An *in situ* breast cancer mouse model was established by injecting 5×10^4^ 4T1 cells into the mammary fat pads of BALB/c mice. The 4T1 cells were transfected with the firefly luciferase reporter gene in order to monitor the tumor progression in real time. An allogeneic HSCT model was then established by transplanting bone marrow mononuclear cells from C57BL/6 mice to the BALB/c mice. To investigate the influence of T lymphocyte proliferation following allogeneic bone marrow transplantation, the levels of CD3^+^CD8^+^ cytotoxic T lymphocytes (CTLs) and CD4^+^CD25^+^ regulatory T cells were determined. In addition, IFN-γ and granzyme B expression levels in splenic lymphocytes were analyzed using flow cytometry. Allogeneic HSCT was found to significantly promote the proliferation and cytotoxicity of CTLs and suppress the growth of breast cancer. Furthermore, the secretory levels of IFN-γ and granzyme B by T cells were elevated following allogeneic HSCT. These results indicated that alloreactive T cells increased the secretion of IFN-γ, which promoted the alloresponse of donor CTLs. In addition, the CTLs produced granzyme B, which exerted a tumor suppressive effect.

## Introduction

Graft-versus-host disease (GVHD), also known as graft-versus-tumor (GVT) effects, has been demonstrated to enhance the therapeutic effect of allogeneic hematopoietic stem cell transplantation (HSCT) in the treatment of leukemia (1). The GVT effects may also observed in patients with solid tumors, including breast, renal and colorectal cancer, treated with allogeneic transplantation (2). The major GVT effectors are cytotoxic T lymphocytes (CTLs), which recognize allogeneic histocompatibility antigens and unique tumor antigens (1). In addition, accumulating evidence indicates that interferon (IFN)-γ exerts a protective effect against GVHD, whilst also participating in the GVT reaction (3,4).

Cellular and molecular mechanisms underlying the GVT effects remain poorly understood. However, it is hypothesized that donor CTLs exhibit a cytotoxic effect against minor histocompatibility antigens or tumor-associated antigens. In addition, regulatory T cells (Tregs) have been demonstrated to protect the host from GVHD by suppressing donor immune cells, and may also block antitumor immune responses (5). IFN-γ has been found to sensitize tumor cells to CTLs, thus, inhibiting the expansion of tumor cells and upregulating the expression of Fas and major histocompatibility complex (MHC) molecules (6). Furthermore, IFN-γ has been hypothesized to function directly on CD8^+^ T cells to stimulate the development of the CTL response (7,8). However, the role of IFN-γ in graft-versus-solid tumor effects remains unclear.

In present study, the mechanisms underlying the immune antitumor reactivity of allogeneic HSCT and the regulation of alloreactive CTLs and Tregs by IFN-γ were investigated. The secretion of IFN-γ, as well as the expansion and cytotoxicity of CTLs, were hypothesized to enhance following allogeneic HSCT; thus, IFN-γ may promote GVT effects by enhancing CTL proliferation and cytotoxicity.

## Material and methods

### Tumor models

Animal studies were performed in accordance with the guidelines from the Nankai University Animal Care and Use Committee (Tianjin, China) and conformed to the National Institutes of Health Guide for Care and Use of Laboratory Animals. Mice (9–12 weeks old, n=18) were purchased from the Experimental Animal Institute of the Chinese Academy of Medical Sciences (Beijing, China) and were housed under standard laboratory conditions. The breast cancer cell line, 4T1 (American Type Culture Collection, Rockville, MD, USA), was transfected with the firefly luciferase (Fluc) reporter gene, and 5×10^4^ 4T1-Fluc cells were *in situ* injected into the mammary fat pads of 9–12 week-old female BALB/c mice.

### HSCT

BALB/c mice recipients were administered autoclaved and acidified water containing antibiotics (30 μg/ml fluconazole and 100 μg /ml norfloxacin; Pfizer, New York, NY, USA) at day 5 prior to and at week 2 following irradiation. HSCT was performed as previously described (9,10). Briefly, at day 6 following the 4T1 cell challenge, mice were irradiated with 9.5-Gy whole-body γ-irradiation in two divided doses that were 2 h apart. For HSCT, the BALB/c mice were injected with 0.2 ml phosphate-buffered saline containing 2.0×10^5^ bone marrow mononuclear cells from female red fluorescent protein (RFP) transgenic C57BL/6-RFP (H-2^b^), C57BL/6 or BALB/c mice via a tail vein injection within 2 h after irradiation.

### Bioluminescence imaging (BLI)

BLI of tumor progression in the living mice was performed as previously described (11,12). Briefly, mice underwent BLI to assess Fluc expression using an *in vivo* Imaging System (IVIS 200; Xenogen Corporation, Hopkinto, MA, USA). Following anesthesia with 2% isoflurane, mice were intraperitoneally injected with 150 mg/kg D-luciferin (Biosynth International, Naperville, IL, USA), and underwent 1 sec to 5 min scans to analyze Fluc expression. The bioluminescence signals were quantified using Living Image Software (Xenogen Corporation).

### Flow cytometric analysis

Fluorescence-activated cell sorting (FACS) analysis of splenocytes was performed using phycoerythrin- or allophycocyanin-conjugated antibodies against mouse CD3 (BD Pharmingen, San Diego, CA, USA), CD8 (eBioscience, San Diego, CA, USA), CD4 (BD Pharmingen), CD25 (BD Pharmingen), IFN-γ (BD Pharmingen) and granzyme B (eBioscience). Cells were labeled in accordance with the manufacturer’s instructions and were analyzed on a FACS Calibur (BD Biosciences, Heidelberg, Germany) using CellQuest software (BD Biosciences).

### Immunofluorescence staining

Tumors were isolated at the end of the imaging studies and sectioned for immunofluorescence staining. Briefly, frozen sections were fixed with 4% paraformaldehyde and incubated with anti-CD8 (BD Pharmingen) or anti-RFP antibodies (Invitrogen Life Technologies, Carlsbad, CA, USA) overnight at 4°C. The sections were washed and incubated with secondary antibodies at room temperature for 30 min, and the cell nuclei were then counterstained with 4,6-diamidino-2-phenylindole (BD Pharmingen). The sections were observed and imaged using a fluorescence microscope (Olympus Corporation, Tokyo, Japan).

### Statistical analysis

Statistics were calculated using SPSS software, version 16.0 (SPSS, Inc., Chicago, IL, USA). Data are expressed as the mean ± standard error of the mean. The statistical significance of intergroup differences of the mean values were analyzed using one-way analysis of variance with Bonferroni’s test. P<0.05 was considered to indicate a statistically significant difference.

## Results

### Labeling 4T1 cells with the Fluc reporter gene

**Figure 1:**
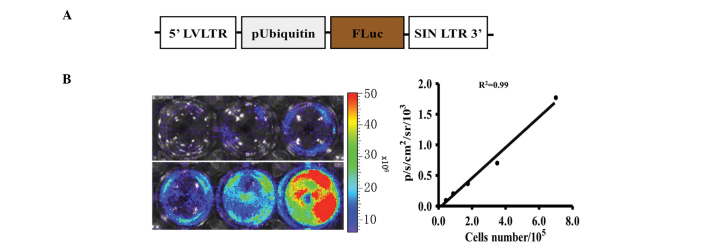
Transduction of 4T1 cells with the Fluc reporter gene. (A) Schematic representation of the Fluc reporter gene. (B) *Ex vivo* imaging analysis of 4T1-Fluc cells demonstrated an increase in the bioluminescence signal intensity correlating with the cell number (r^2^=0.99). Fluc, firefly luciferase.

An imaging platform for tracking 4T1 cells in mice was developed using the Fluc reporter gene (Fig. 1A). Following culture in 24-well plates, a strong correlation (r^2^=0.99) was observed between the Fluc signal intensity and the cell number *ex vivo* (Fig. 1B).

### HSCT significantly suppresses tumor growth

**Figure 2:**
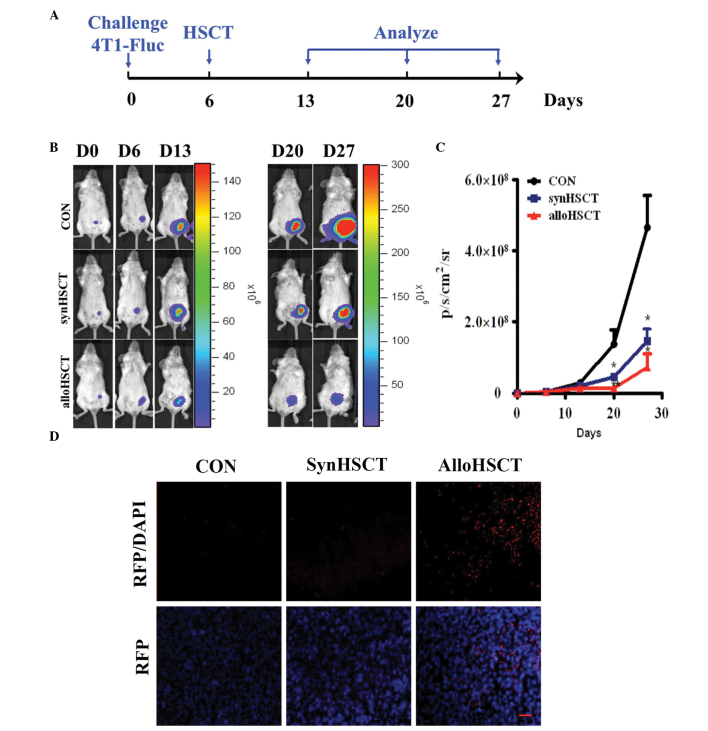
Allogeneic HSCT significantly suppressed the growth of breast cancer tumors. (A) Mouse model and treatment schedule of HSCT. (B) BLI of representative mice from each group are shown following 4T1-Fluc cell inoculation. (C) Quantitative analysis of the BLI signal intensity in each group. Bioluminescence activity is shown as photons/sec/cm^2^/sr and results are expressed as the mean ± standard error of the mean (≥5 mice per group). ^*^P<0.05 and ^**^P<0.01, vs. control. (D) Breast cancer tissues from the BALB/c mice in the Con, SynHSCT and AlloHSCT groups were obtained and stained for RFP on day 35. The nuclei were counterstained with 4,6-diamidino-2-phenylindole (scale bar = 50 μm). HSTC, hematopoietic stem cell transplantation; Con, control group; SynHSCT, syngeneic HSCT group; alloHSCT, allogeneic HSCT group; RFP, red fluorescent protein; BLI, bioluminescence imaging; Fluc, firefly luciferase.

To investigate the potential GVT effects of allogeneic HSCT, an *in situ* breast cancer model in BALB/c mice was used. Following total body irradiation with 9.5 Gy on day 6, bone marrow mononuclear cells from allogeneic C57BL/6 or syngeneic BALB/c donors were transplanted into BALB/c mice (Fig. 2A). The development of tumors was analyzed using BLI of Fluc. The average Fluc signal intensity increased rapidly in the control group that did not receive treatment (Fig. 2B and C). By contrast, the Fluc signal in the allogeneic HSCT group increased slightly and statistically significant differences were observed between the two groups (P<0.05 on day 27; P<0.01 on day 20). Immunofluorescence staining of RFP in the tumor tissues demonstrated that there were a large number of infiltrated tumor cells, which were derived from the donor bone marrow hematopoietic stem cells following allogeneic HSCT (Fig. 2D).

### IFN-γ, secreted by T lymphocytes, mediates antitumor effects

**Figure 3:**
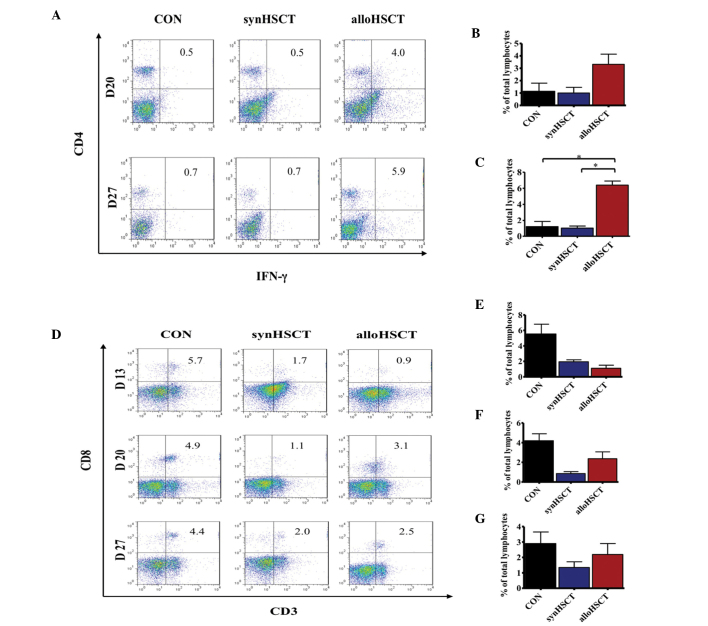
IFN-γ secreted by T lymphocytes mediates antitumor effects following allogeneic HSCT. The spleens of mice receiving HSCT were harvested on days 20 and 27 for analysis of IFN-γ expression in CD4^+^ T cells. Representative flow cytometry plots demonstrate the percentage of (A) CD4^+^ T cells expressing IFN-γ. Results are expressed as the mean ± SEM (n=3 per group). Quantitative analyses show the number of CD4^+^ T cells expressing IFN-γ on days (B) 20 and (C) 27. (D) At the indicated time points, lymphocytes in the spleens of mice receiving HSCT were obtained and the levels of CD3^+^CD8^+^ T cells were analyzed by flow cytometry. IFN, interferon; HSCT, hematopoietic stem cells transplantation; Con, control group; SynHSCT, syngeneic HSCT group; alloHSCT, allogeneic HSCT group; SEM, standard error of the mean.

IFN-γ has been previously shown to be involved in allogeneic GVT effects and the inhibition of GVHD (13). To elucidate the mechanism of GVT, splenic lymphocytes were intracellularly stained for IFN-γ and analyzed using flow cytometry (Fig. 3). Following allogeneic HSCT, the production of IFN-γ in CD4^+^ lymphocytes was significantly increased compared with the control and syngeneic HSCT groups on day 20 and 27 (Fig. 3B and C).

**Figure 4:**
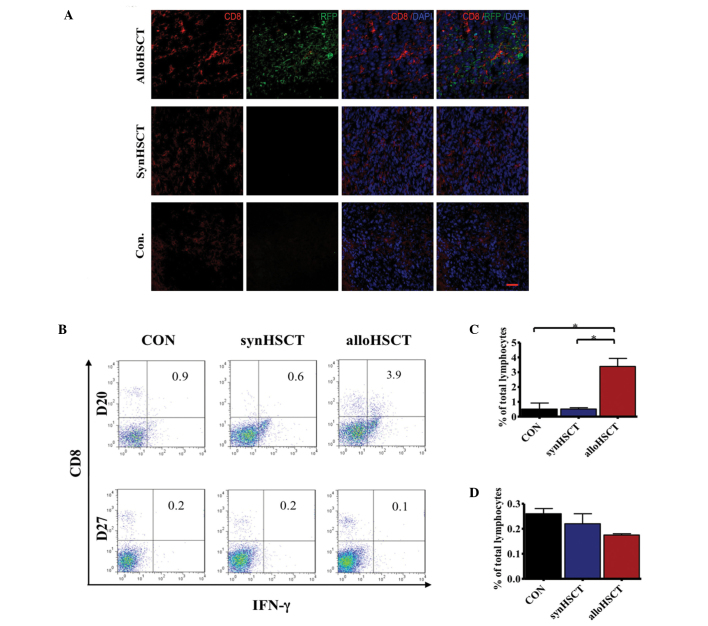
IFN-γ secreted by T lymphocytes mediates antitumor effects following allogeneic HSCT. (A) Breast cancer tissues of BALB/c mice from the Con, SynHSCT and AlloHSCT groups were obtained and stained for CD8 on day 35. The nuclei were counterstained with 4,6-diamidino-2-phenylindole (scale bar = 50 μm). (B) Quantitative analyses show the number of CD8^+^ T cells expressing IFN-γ. CD8^+^ T cells producing IFN-γ on days (C) 20 and (D) 27 (^*^P<0.05). Data are presented as the mean ± SEM (n=3 per group). IFN, interferon; HSCT, hematopoietic stem cells transplantation; Con, control group; SynHSCT, syngeneic HSCT group; alloHSCT, allogeneic HSCT group; SEM, standard error of the mean.

In addition, CTLs have been previously demonstrated to play an important role in the GVT effects (14), and were shown to be regulated by CD4^+^ T helper cells secreting IFN-γ. To determine whether the proliferation of CTLs following allogeneic HSCT is different compared with syngeneic HSCT in breast cancer animal models, CD3^+^CD8^+^ CTLs were analyzed by flow cytometry (Fig. 3D). On days 13, 20 and 27, the mice were euthanized by intrapertioneal injection of 4% chloral hydrate and single cell suspensions of splenic lymphocytes were obtained. Flow cytometric analysis revealed that the number of CTLs following allogeneic HSCT was higher compared with syngeneic HSCT on days 20 and 27 (Fig. 3E–G), indicating that despite the number of CTLs following transplantation not completely recovering to a normal level, the IFN-γ secreted by T lymphocytes following allogeneic HSCT may promote the expansion of CTLs and exert a suppressive effect on cancer. To further investigate the long-term GVT effects mediated by CTLs, tumor tissues were isolated at day 40 following tumor cell inoculation. CD8 expression in the tumor tissues was found to be significantly enhanced in the allogeneic HSCT group compared with the syngeneic HSCT group (Fig. 4A).

Furthermore, IFN-γ expression levels in CD8^+^ lymphocytes (Fig. 4B) increased on day 20, returning to the basal level by day 27, in mice treated with allogeneic transplantation (Fig. 4C and D). These results provide novel insights into the role of IFN-γ in regulating GVT-associated alloreactivity of allogeneic HSCT.

### Proliferation of Tregs is suppressed following allogeneic HSCT

**Figure 5:**
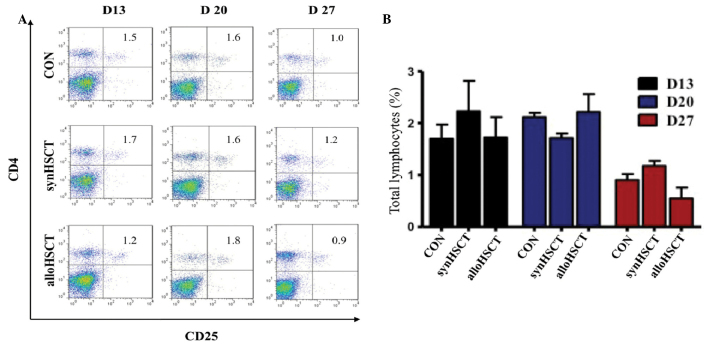
Proliferation of Tregs was suppressed following allogeneic HSCT. (A) Spleen cells of mice (n=3 per group) from the Con, SynHSCT and AlloHSCT groups were prepared and analyzed for CD4^+^CD25^+^ Tregs using flow cytometry. (B) Percentages of CD4^+^CD25^+^ Tregs in spleen lymphocytes at the indicated time points. Results are expressed as the mean ± standard error of the mean. HSCT, hematopoietic stem cells transplantation; Con, control group; SynHSCT, syngeneic HSCT group; AlloHSCT, allogeneic HSCT group; Treg, regulatory T cells.

To examine the effect of allogeneic HSCT on Tregs, splenic lymphocytes were isolated from mice treated with allogeneic or syngeneic HSCT, and the levels of CD4^+^CD25^+^ Tregs were analyzed (Fig. 5A). No significant differences were observed among the levels of Tregs in the control, syngeneic HSCT and allogeneic HSCT groups on days 13, 20 and 27 (Fig. 5B), indicating that the GVT effects may be mediated by enhancing CTL activity, as opposed to suppressing Tregs.

### CTLs produce granzyme B and exert antitumor activity following allogeneic HSCT

**Figure 6:**
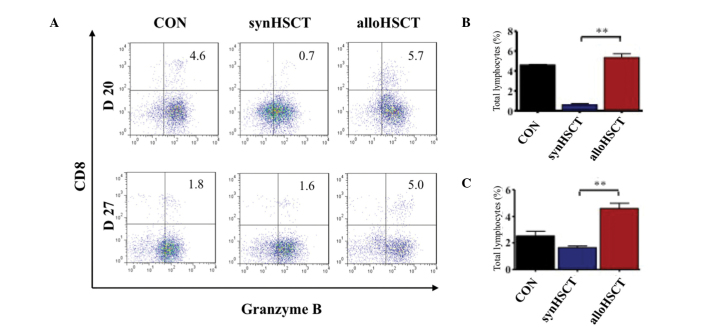
CTLs produce granzyme B and exert antitumor activity following allogeneic HSCT. (A) Granzyme B production in CD8^+^ CTLs was determined using intracellular cytokine staining (n=3 per group). Quantitative analysis of the expression of granzyme B in CTLs at days (B) 20 and (C) 27 (^**^P<0.01). HSCT, hematopoietic stem cells transplantation; Con, control group; synHSCT, syngeneic HSCT group; alloHSCT, allogeneic HSCT group; CTLs, cytotoxic T lymphocytes.

To verify the mechanism of CTL-mediated cytotoxicity and apoptosis, splenic lymphocytes were analyzed using intracellular staining of granzyme B. CD8^+^ T lymphocytes were shown to produce granzyme B (Fig. 6A). Furthermore, the number of granzyme B^+^ CTLs following allogeneic HSCT was greater compared with syngeneic HSCT, with statistically significant differences (P<0.01) observed on days 20 and 27 (Fig. 6B and C, respectively). Thus, the results indicated that CTLs secrete granzyme B and may mediate the apoptosis of target cells following allogeneic HSCT.

**Figure 7:**
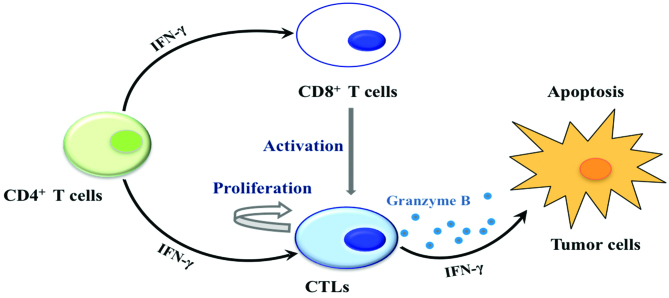
Putative model outlining the IFN-γ signaling pathway mediating the GVT effects of allogeneic HSCT. HSCT, hematopoietic stem cells transplantation. IFN, interferon; GVT, graft-versus-tumor; CTL, cytotoxic T lymphocyte.

Considering the significant inhibition of tumors following allogeneic HSCT, GVT was found to be mediated by donor CTL cytotoxicity via IFN-γ and granzyme B. Furthermore, the activation and proliferation of CTLs was enhanced by IFN-γ, which was secreted by CD4^+^ T cells (Fig. 7).

## Discussion

In the present study, the immune mechanism of GVT activity in allogenenic HSCT was investigated. The allograft was demonstrated to confer significant GVT activity against breast cancer. Furthermore, the results revealed that GVT effects are dependent on the IFN-γ and granzyme B signaling pathways. Allotransfer was demonstrated to mediate the expansion of CTLs, resulting in higher levels of granzyme B. In addition, the expansion of Tregs, which is associated with tumor progression, was significantly suppressed at the later stages following allogeneic transplantation. Therefore, these observations indicate that alloreactive CD4^+^ and CD8^+^ T cells produce IFN-γ, which regulates the alloresponse of donor CTL cells, resulting in cytotoxic effects by CTLs on tumor cells.

IFN-γ produced by donor T cells has an important role in the induction of the CD8^+^ T cell-mediated GVT reaction, which results in tumor delay or even tumor rejection in the absence of lethal GVHD (15). The ability of IFN-γ to inhibit the growth of tumor cells (16,17), including breast cancer cells (18), has been demonstrated in several studies. In breast cancer patients with skin metastasis, *in situ* injection of IFN-γ resulted in total or partial regression of the skin lesions (19). A number of studies have hypothesized that IFN-γ exerts its antitumor effects by regulating several aspects of innate and adaptive immunity, including activating CTLs and natural killer cells and inhibiting the generation of CD4^+^CD25^+^ Tregs (6,20,21). The results of the present study revealed that the expansion or activation/proliferation of CTLs was slightly increased following allogeneic transplantation, as compared with syngeneic transfer, and allogeneic transplantation suppressed the expansion of Tregs at a later stage. CTLs have been shown to be associated with a positive outcome (improved effect on suppression of tumor growth than that of Tregs) (22), whilst immunosuppressive Tregs are associated with tumor progression and poor prognosis (23); thus, the response to allotransfer therapy correlates with inverse changes in the numbers of CTLs and Tregs.

Despite CTLs exerting an efficient tumor rejection effect following allogeneic transplantation, the proportion of CTLs was lower in mice treated with HSCT compared with mice that received no treatment. Thus, the regulation of T cells by IFN-γ alone may be insufficient to explain the GVT effects of allogeneic HSCT, and other mechanisms may be involved. IFN-γ signaling in tumor cells inhibits cancer cell expansion by inducing apoptosis and suppressing proliferation, and may also increase the sensitivity of tumor cells to the cytolytic activity of alloreactive CTLs via the upregulation of MHC class I and Fas molecules (24). The results of the present study revealed high levels of granzyme B production by CD8^+^ CTLs. Granzyme B is known to be delivered by perforin into target tumor cells, where it induces apoptosis by cleaving critical substrates (25). The execution of apoptosis by granzyme B usually occurs via the activation of the caspase family of cysteine proteases, which are central regulators of the apoptotic pathway for multiple inducers of cell death (26). Contact-dependent lysis is also critical for alloreactive CTLs to mediate GVT effects in allogeneic HSCT recipients.

The majority of target antigens for the GVT effects have yet to be identified, but include unique tumor antigens and host alloantigens. Allogeneic immunotherapy for cancer is restricted by suboptimal GVT effects and GVHD associated with GVT effects. Previous studies have demonstrated that GVT effects and GVHD may be separated by IFN-γ in hematopathy and solid tumor disease animal models (4,27). GVHD- and GVT-associated alloresponses of CD8^+^ T cells may be dissociated by an IFN-γ-dependent mechanism, whereby CD8^+^ T cells from IFN-γ receptor-deficient donor mice induced more severe GVHD in allogeneic recipients compared with transfer cells from wild-type donors.

In conclusion, alloreactive T cells produce IFN-γ, which promotes the alloresponses of donor CTLs. In addition, granzyme B, produced by CTLs, exerts a tumor suppressive effect. Exploitation of these immunotherapy mechanisms may improve the outcome of clinical bone marrow transplants and lead to the development of novel treatments for breast cancer, as well as other solid tumors.
